# Retraction: Extracellular vesicles carry lncRNA SNHG16 to promote metastasis of breast cancer cells via the miR-892b/PPAPDC1A axis

**DOI:** 10.3389/fcell.2024.1445363

**Published:** 2024-06-14

**Authors:** Frontiers Editorial Office

The Journal retracts the 2021 article cited above.

Following publication, concerns were raised regarding the validity of the data in the article. An investigation by our internal Research Integrity department, conducted in accordance with Frontiers policies, was unable to confirm the validity of the data presented and we cannot vouch for its reliability. The article is therefore retracted.

This retraction was approved by the Chief Executive Editor of Frontiers. The authors do not agree to this retraction.

